# Contained Rupture of a Small Mycotic Abdominal Aneurysm in a Patient With Infective Endocarditis

**DOI:** 10.7759/cureus.18963

**Published:** 2021-10-22

**Authors:** Onoriode Kesiena, Rafael Carlos Da Silva, Navin Kumar

**Affiliations:** 1 Internal Medicine, Piedmont Athens Regional Medical Center, Athens, USA

**Keywords:** aortic, endocarditis, mycotic, rupture, aneurysm

## Abstract

A contained ruptured mycotic abdominal aneurysm is one of the complications of infective endocarditis. It is a complication that physicians should entertain when patients with infective endocarditis present with a complaint of back pain. This case report aims to increase the awareness of the possibility of a rupture of a small size abdominal mycotic aneurysm. This is a 36-year-old female with a history of intravenous (IV) drug use and infective endocarditis secondary to methicillin-sensitive *Staphylococcus aureus* presented with acute right-sided lower back pain. Work-up revealed a contained ruptured 2.5 cm mycotic abdominal aneurysm. She had an open surgical repair of the abdominal aorta followed by a mitral valve replacement a week later and she was discharged home on antibiotics and an anticoagulant. Untreated, a mycotic aneurysm can expand quickly and has a higher likelihood of rupturing as compared to an atherosclerotic abdominal aortic aneurysm. A contained ruptured mycotic abdominal aneurysm can lead to a dramatic hemodynamic compromise when it becomes uncontained, hence it is prudent that it is acted after it is diagnosed. Most authors recommend prompt surgery for all patients irrespective of the size of the aneurysm. Younger age is a factor to consider in choosing a repair approach despite the complications associated with both open surgical and endovascular repair.

## Introduction

Mycotic aortic aneurysm (MAA) is a focal dilation of the aorta due to an infection [1] . It is a rare condition with an incidence of around 0.65%-2% of all aortic aneurysms in western countries [2,3]. The typical infectious cause arises from a distant infection such as the heart, through either bacteremia or septic embolization [4]. In infective endocarditis, septic embolization is typically secondary to pathogens such as *Staphylococcus aureus* and *Salmonella *spp [5,6]. The pathophysiology involves an infection of the vasa vasorum which causes inflammation and ischemia of the vessel wall leading to progressive dilation of the infected segment resulting in a contained rupture and a pseudoaneurysm [7]. Currently, the literature comments on the increased risk of rupture of a mycotic aneurysm due to a rapid increase in size and inflammatory pathology. This case report increases the awareness of the rupture of a small-size MAA.

## Case presentation

A 36-year-old female with a history of IV drug use, recent MSSA bacteremia, and bacterial endocarditis of a native mitral valve complicated by a septic embolus to the brain with residual left-sided weakness presented to the emergency room for evaluation of worsening right-sided lower back pain, which started 10 days prior to presentation. The pain was dull in nature and was worse on waking up in the morning and better with ibuprofen. It did not radiate and at the time of presentation, and the severity was 7/10. She denied trauma, similar previous episodes, fever, urinary or fecal incontinence. She had left against medical advice at another facility 10 days prior to presentation, where she was managed for endocarditis. She had a significant smoking and alcohol consumption history. She had a history of allergy to erythromycin, penicillin, and azithromycin.

On examination, blood pressure 121/80 mmHg, pulse 123 bpm, temperature 97.9 ˚F, respiratory rate 22 cpm, oxygen saturation 99%. Significant physical exams included tachycardia, tenderness at the umbilical region, right paraspinaland lumbar tenderness, and left-sided weakness. Table 1 shows the initial laboratory investigation. 

**Table 1:** Initial laboratory investigation.

Laboratory	Results
White cell count	14,800/mm^3^
Neutrophils	83%
Lymphocytes	7%
Monocytes	10%
Hemoglobin	10.8 g/dL
Platelets	401,000/mL
Sodium	138 mmol/L
Potassium	3.3 mmol/L
Chloride	103 mmol/L
Bicarbonate	23 mmol/L
Creatinine	0.85 mmol/L
Blood urea nitrogen	9 mmol/L
Urinalysis	Trace of leukocytes
Blood cultures	No growth after five days

**Figure 1:**
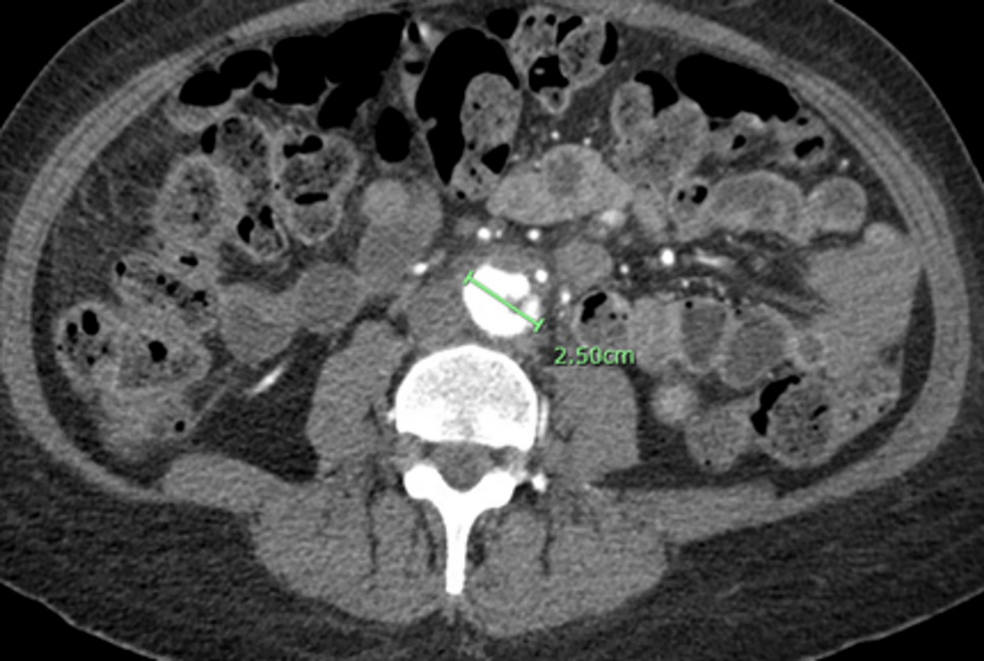
Cross-sectional CT abdomen with contrast showing 2.5 cm contained ruptured mycotic abdominal aneurysm

**Figure 2:**
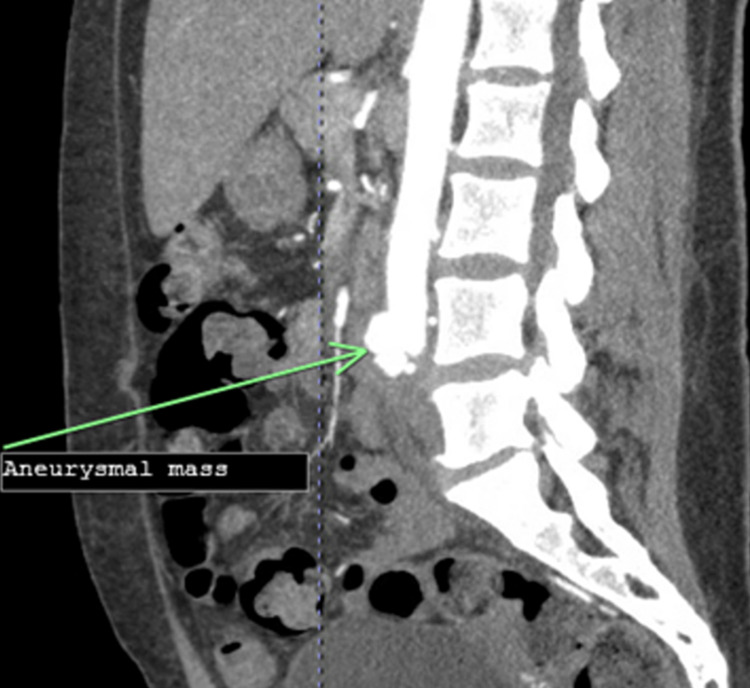
Sagittal view of the mycotic abdominal aneurysm

**Figure 3:**
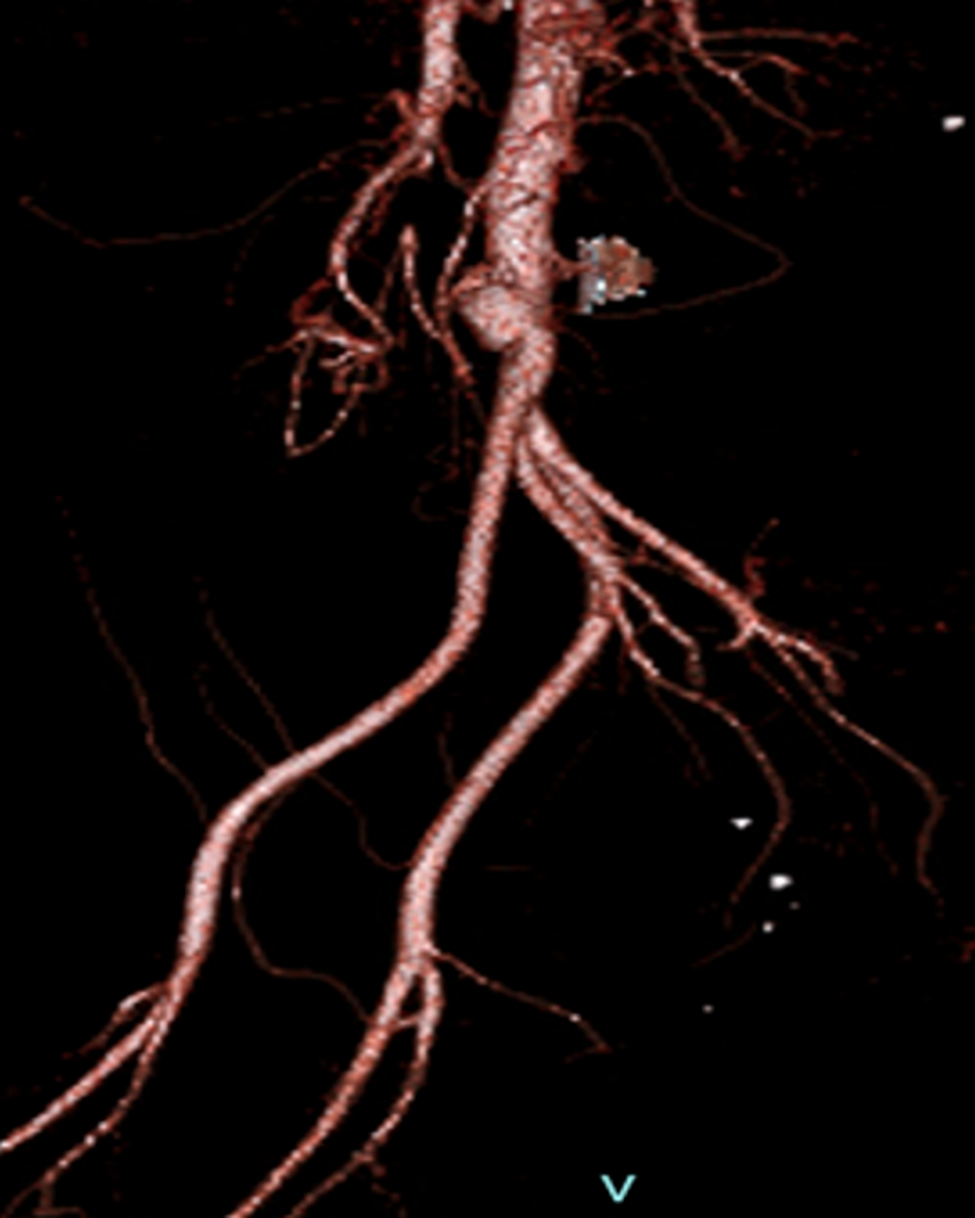
A 3-D reconstituted imaging from CT scan with contrast showing mycotic infra-renal aneurysm

An electrocardiogram showed sinus tachycardia. An echocardiogram showed an ejection fraction of 59%. Mitral valve endocarditis with a large (2cm), mobile vegetation was noted on the posterior leaflet with associated moderate mitral regurgitation. The vegetation extended posteriorly into the left atrium during ventricular systole. Angio tomography (CTA) chest/abdomen/pelvis showed a lobulated irregular pseudoaneurysm of the aortic bifurcation measuring up to 2.5 cm with involvement of the proximal aspect of the common iliac arteries. In addition, there was a short segment occlusion of the distal superior mesenteric artery (SMA) branch of about 1.6 cm (Figures 1-3). The impression was consistent with a mycotic aneurysm with irregular lobular contours at the aortic bifurcation compatible with contained rupture.

She was started on morphine, ondansetron, vancomycin, and cefepime, and vascular surgery was consulted. In addition, cardiology, cardiothoracic surgery, and infectious disease assisted with her care. She had an open surgical aortic aneurysm repair with an intraoperative finding of a phlegmonous mass with heme stain seen at the aortic bifurcation with an inflammatory reaction around the aorta extending to the level of the inferior mesenteric artery. Pathology specimen showed acute inflammatory changes of the resected aortic segment. A week later, she underwent mitral valve replacement surgery with a 29-mm bioprosthetic valve. She was discharged home on an anticoagulant, nafcillin, hydromorphone, aspirin, and atorvastatin. 

## Discussion

The association between infective endocarditis and MAA is well established. However, this is a rare complication, posing difficulty in establishing clear guidelines in terms of its management as in other rare diseases. According to Sörelius [3], there is a need to standardize the definition, terminology, diagnostic criteria, and further classification of MAAs. Our case report aimed to add to the literature, describing a full picture in terms of risk factors, clinical presentation, morphological aspects, and treatment implemented to help in the standardization of this particular entity.

In the last 15 years, the MAA diagnostic has been based on a variety of combinations of the following four criteri40a: (1) clinical presentation; (2) laboratory findings; (3) radiological, and (4) intraoperative findings. Based on those criteria, our patient presented with infective endocarditis, fever, back pain, and dilation of 2.5 cm of the aortic at the level of the iliac arteries bifurcation.

In a systematic review, MAA was found to develop anywhere in the aorta, being 60% located in the infrarenal aorta, with only 2% located at the aortic bifurcation [8]. Similarly, our patient presented with a distal lesion at the level of the aortic bifurcation into the iliac arteries. The mortality is extremely high ranging between 26% and 44% [8]. Fortunately, our patient had a successful surgery, which led to a good outcome, which could be explained by her youthful age. Also, in the above-mentioned systematic review involving 963 patients, the mean patient age was 69 years with 73% being men. Our patient could be considered an outlier in this sample, given her female gender and youthful age.

The management is based on a prolonged antibiotic, culture-guided therapy, and open surgical or endovascular approach. There are no guidelines in the literature in terms of the size of the aneurysm that might guide the decision-making about open surgery versus endovascular therapy (EVT). According to the American Heart Association, for most patients with MAA, resection and in situ revascularization is reasonable. On the other hand, EVT can be an option in unstable patients, with severe comorbidities and in those with uncontrolled bleeding with aorto-enteric fistula [9]. Our patient was stable on admission and the MAA was infrarenal located.

## Conclusions

In conclusion, a small-sized mycotic abdominal aneurysm has a higher likelihood of rupture as compared to an atherosclerotic abdominal aortic aneurysm. Due to this risk of a rapid rupture, the diagnoses must be prompt and managed surgically. Clinical factors which contributed to a favorable outcome are younger age and hemodynamic stability at diagnosis. This report adds to the increasing literature on the clinical characteristics of a contained ruptured mycotic abdominal aneurysm and a successful treatment option.
